# Eye accommodation-inspired neuro-metasurface focusing

**DOI:** 10.1038/s41467-023-39070-8

**Published:** 2023-06-06

**Authors:** Huan Lu, Jiwei Zhao, Bin Zheng, Chao Qian, Tong Cai, Erping Li, Hongsheng Chen

**Affiliations:** 1 Interdisciplinary Center for Quantum Information, State Key Laboratory of Extreme Photonics and Instrumentation, ZJU-Hangzhou Global Scientific and Technological Innovation Center, Zhejiang University, Hangzhou, 310027 China; 2 International Joint Innovation Center, The Electromagnetics Academy at Zhejiang University, Zhejiang University, 314400 Haining, China; 3 Key Lab of Advanced Micro/Nano Electronic Devices & Smart Systems of Zhejiang, Jinhua Institute of Zhejiang University, Zhejiang University, 321099 Jinhua, China; 4 Shaoxing Institute of Zhejiang University, Zhejiang University, 312000 Shaoxing, China; 5 School of Electronic Science and Engineering, Nanjing University, 210023 Nanjing, Jiangsu Province China; 6 The Air and Missile Defend College, Air force Engineering University, 710051 Xi’an, China

**Keywords:** Metamaterials, Optical materials and structures

## Abstract

The human eye, which relies on a flexible and controllable lens to focus light onto the retina, has inspired many scientific researchers to understand better and imitate the biological vision system. However, real-time environmental adaptability presents an enormous challenge for artificial eye-like focusing systems. Inspired by the mechanism of eye accommodation, we propose a supervised-evolving learning algorithm and design a neuro-metasurface focusing system. Driven by on-site learning, the system exhibits a rapid response to ever-changing incident waves and surrounding environments without any human intervention. Adaptive focusing is achieved in several scenarios with multiple incident wave sources and scattering obstacles. Our work demonstrates the unprecedented potential for real-time, fast, and complex electromagnetic (EM) wave manipulation for various purposes, such as achromatic, beam shaping, 6 G communication, and intelligent imaging.

## Abstract (web summary)

Here the authors propose for the first time the concept of supervised-evolving learning (SEL) and a corresponding SEL-driven adaptive focusing (SELAF) system. This metasurface can adaptively realize focusing at any specified position for waves incident from any direction. This work demonstrates unprecedented potential for tasks involving real-time, fast and complex electromagnetic wave manipulation.

## Introduction

Light focusing has been a long-standing research topic for centuries. Optical focusing lenses are ubiquitous in our daily lives and modern optical laboratories. For example, digital cameras perceive image information by relying on in-built complex optical lenses systems^1^. They are typically manufactured on various bulky substrates, generating user-defined beam intensity profiles or beam shapes by rationally designing microstructures^2^. Significant effort has been made with the advent of metasurfaces to miniaturize and integrate optical lenses. Metasurfaces are artificially engineered structures composed of subwavelength scatterers with periodic or quasi-periodic arrangements^3–6^. By carefully designing a subwavelength element and spatiotemporal layout, many attractive optical responses can be customized at will^7–13^. Among many functional devices, a metalens is one of the most thought-provoking. Compared to conventional bulky lenses, which specifically rely on the polished surface profile of transparent optical materials to attain the required gradual phase change, metalenses can focus incident light with more compact dimensions.

**Fig. 1:**
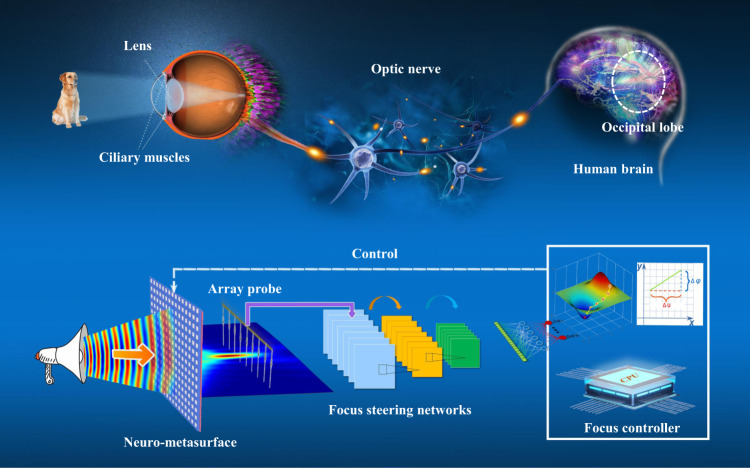
Mimicking eye accommodation with the SELAF neuro-metasurface. Through the optic nerve, the electrical signals stimulated by visible radiation are transmitted to the occipital lobe, where they are interpreted as vision^33^. Neural signals, originating from the oculomotor nerves, travel through the parasympathetic nervous system and travel via the short ciliary nerves. Then, the contraction of the ciliary muscles alters the focal distance, controlling the ability to focus nearer or farther images on the retina (as shown in the upper half of the diagram). Analogously, a metasurface first modifies the transmissive phases and amplitudes of incident waves, and an array of monopole probe detects these EM waves. The detected signals subsequently pass through the convolutional layers and fully connected layers of a focus steering network and are thus converted into focus steering signals. Then, a focus controller generates voltage regulation decisions based on both the steering signals and prior knowledge. Thereafter, the control circuit applies the generated decisions to the metasurface to adjust the states of the diodes, thereby changing the entire transmitted EM spectrum for subsequent focusing (as shown in the bottom half of the diagram).

In the past decade, we have witnessed numerous studies on metalenses using various metasurface structures with the goal of achieving broadband, achromatic performance, high efficiency, and more^14–21^. Most metalenses work only in predefined environments, which makes it challenging to cater to the ever-changing application scenarios and incident waves. Therefore, an intelligent adaptive strategy is required. Despite fruitful studies on adaptive optics by empowering artificial intelligence (deep learning) on metasurfaces^22–29^, the success of deep learning relies heavily on the quantity and quality of available training data and requires prior information about the environment. Deep learning may fail when confronted with rapidly changing environments during a focusing task. Given these factors, a practical approach to avoid time-consuming searches and develop self-adaptability is highly sought after, although challenging. The human eye is a perfectly focused system; more than 80% of the information about our surroundings is acquired through our eyes^30^. In the eye, the lens primarily focuses the incident light onto the retina and achieves a wide focal range by adjusting its shape via accommodation^31^. Therefore, the visual system is highly adaptive and responsive to environmental changes^32,33^. Suppose a naturally intelligent focusing system can be designed to automatically converge the EM waves (light) in different environments, such as the human eye. In that case, this will significantly simplify the design of the device and facilitate its application (Fig. 1).

In this work, inspired by the accommodation of human eyes, we propose the concept of supervised evolving learning (SEL) and demonstrate an SEL-driven adaptive focusing (SELAF) neuro-metasurface. Without requiring prior knowledge of the complex EM environment, the proposed SELAF system can adaptively converge incident waves to a user-defined position, even in the presence of multiple wave sources or scattering obstacles. This remained effective for multiple wavelengths. In contrast to typical deep-learning methods, the SEL algorithm gradually approaches the focal point by continuously adjusting the voltage on the metasurface. This approach can effectively address the challenges of model bias, environmental dynamics, and diode characteristic uncertainty that are often encountered in deep learning techniques. A series of experiments were performed with different incident angles, dual incident wave sources, and obstacle blocking to verify the proposed SELAF neuro-metasurface. Both the simulated and experimental results show that the SELAF neuro-metasurface exhibits effective and powerful adaptive focusing ability in response to a complex EM environment. The SEL architecture could pave the way for various manipulations of EM waves. It can be extended to scenarios such as 6 G channel enhancement, wireless charging, and EM spectrum imaging.

## Results

Figure 1 illustrates the eye-focusing mechanism and the corresponding equivalence of the proposed SELAF neuro-metasurface. When the incident light reaches the eye, the human brain continuously adjusts the lens until it is focused on the retina. Similarly, the network acquires signals using the array probe and adjusts the metasurface through the controller to achieve focusing at different positions. Essentially, the phase shift produced by each unit cell of the SELAF neuro-metasurface is adjusted in response to the incident wave by changing the voltage applied to the diodes of the neuro-metasurface. Thus, focusing is achieved through successive iterations of voltage alteration (see Methods).

**Fig. 2:**
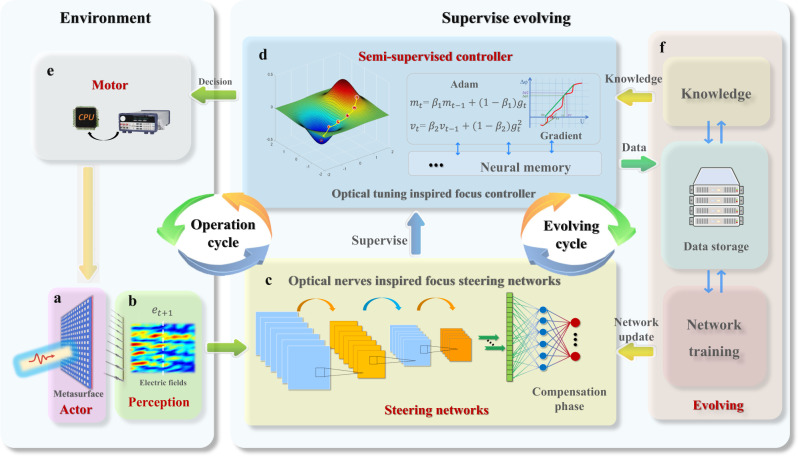
SEL architecture. There are two main components of SEL: one for interaction with the environment and the other for decision-making. **a** The actor is a device that modifies the surrounding EM environment. **b** The perception module transforms the environmental state into an internal representation. **c** The steering network provides supervision for achieving goals. **d** The semi-supervised controller generates decisions in accordance with the supervised parameters from the steering network and local knowledge. **e** The motor, which executes the decisions generated by the controller, drives the actor to change its state to, in turn, affect the environment. **f** The evolutionary module evolves the steering network and the stored local knowledge based on the data collected during operation.

The complete concept of SEL is illustrated in Fig. 2. It consists of two cycles: an operation cycle and an evolution cycle. The proposed SELAF system is a concrete example of an operational cycle in an SEL. Unlike typical supervised learning approaches that output a determined value simultaneously, the SEL algorithm performs multiple steps to achieve an adaptive focus. This exploratory trial-and-error learning method enhances the robustness and adaptability of the metasurfaces in unfamiliar or unknown environments. During the operational cycle, the system interacts with the environment to generate historical data. First, the neuro-metasurface (Fig. 2a) changes the state of its environment (EM field) using its properties. A perception module (array probe, Fig. 2b) translates the environmental state into an internal representation (energy matrix). A steering network (Fig. 2c) performs a supervised parameter prediction (phase compensation). The gradient of the voltage phase shift of the metasurface was obtained (Fig. 2d). By combining the predicted values with local knowledge (Fig. 2f), the controller generates the final decision (metasurface voltage). Finally, a motor (multichannel power supply; Fig. 2e) executes the decision and changes the state of the neuro-metasurface to achieve the task goal. The motor and actor can sometimes be unified, making decisions, and simultaneously influencing the environment. Typically, the steering network provides a target for adjustment, and based on this target, the controller provides a specific execution decision. In the evolving cycle, the compensation phase can be transformed into the metasurface adjustment voltage, which is determined by both the gradient of the voltage phase shift and the corresponding continuous optimizing algorithm (ADAM). Simultaneously, the evolving cycle periodically updates the steering network, thereby improving the performance and efficiency of the actions of the motor.

The SEL employs an evolving circle with three submodules to respond quickly to changing environments: knowledge, data storage, and network training. The data storage submodule stores both the simulated and experimental data collected during the operation cycle. The training submodule retrains the steering network, using the stored data to accelerate the iterative process and enhance the adaptation of the model (see “Methods”). A knowledge submodule then provides decision-making guidance to the controller (see “Methods”). With the accumulation of experimental data, the initial simulation model continued to evolve as it was periodically retrained, significantly improving its environmental adaptability and accelerating the termination of the iterative process.

**Fig. 3:**
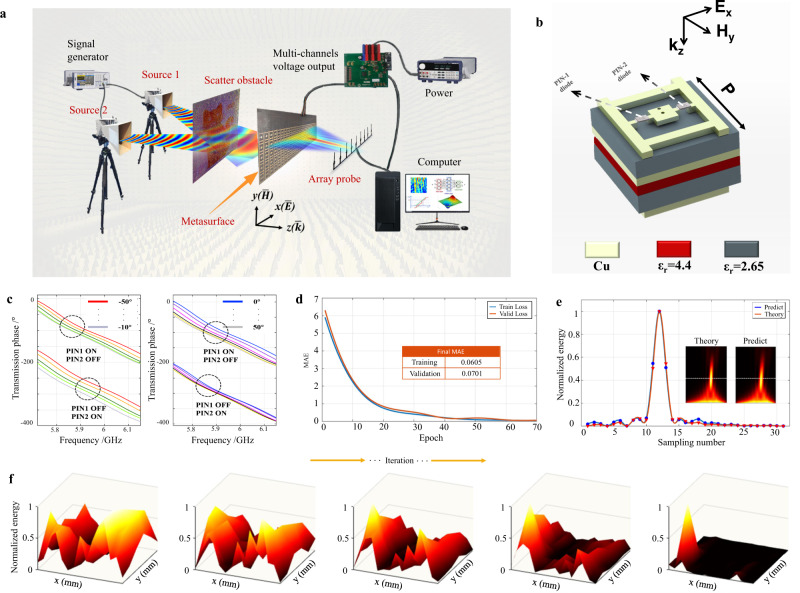
Experimental setup, process, and unit cell properties. **a** Experimental setup. **b** Schematic of the unit cell. **c** Phase distributions at different frequencies and incidence angles. **d** Loss function. Mean average error, MAE. **e** Theoretical and network-predicted electric field results. The curves represent the one-dimensional data at the focal point. **f** Focusing process at random angle incidence. The length and width correspond to the range of the swept field, and the height represents the normalized focusing energy.

The prototype of the SELAF system is illustrated in Fig. 3a. The size of the included metasurface is $100\times 372\times 2.2m{m}^{3}$ ($5\times 31$ unit cells). Each column of units along the y-axis direction shares the same bias voltage, and a switching diode is soldered to the metal on the dielectric substrate. The transmitting antenna generates EM wave signals from a random direction, and the array probe continuously collects one-dimensional electric field data at the specified focusing plane and then transmits them to the SEL system. Subsequently, SEL is applied to predict a series of voltages to regulate the neuro-metasurface (Supplementary Note 1 gives the details of the power supply system). Figure 3b shows a schematic of the designed metasurface unit cell, which has a stacked structure based on PIN-diodes. Specifically, the unit cell is composed of double-layer F4B materials and two PIN diodes are embedded on top to enable 180° phase reversal for incident waves (more details of the unit cell design are provided in Supplementary Note 2). The transmission coefficient of the unit cell is above −0.8 dB over a frequency band of 5.78–6.03 GHz, and the average transmittance over 95%. Such transmission coefficient still holds even when the incidence angle varies between −50° and 50° (see Supplementary Note 2). Although there are only two phase-states for each meta-atom (Fig. 3c), a metasurface composed of 31 unit cells can still enable a high degree of freedom to produce a satisfactory focusing effect, while also supporting focusing at other frequencies (Supplementary Note 3). Figure 3d shows the loss function training results, which indicate that mean absolute error (MAE) losses on the training and test sets are both close to 0. Hence, we conclude that the pre-trained focus steering network is credible. Figure 3e shows the electric field distributions at the probe when the theoretically calculated and predicted voltages were applied to the metasurface. The curves show a one-dimensional electric field energy distribution, whereas insets show a two-dimensional electric field energy at the focal plane. The network could perfectly predict the phase compensation of every unit cell. In Fig. 3f, we illustrate the iterative process of focusing in a demo experiment (the corresponding two-dimensional electric field was remeasured). It can be observed that the energy gradually becomes concentrated at the desired focal position after a series of iterations.

**Fig. 4:**
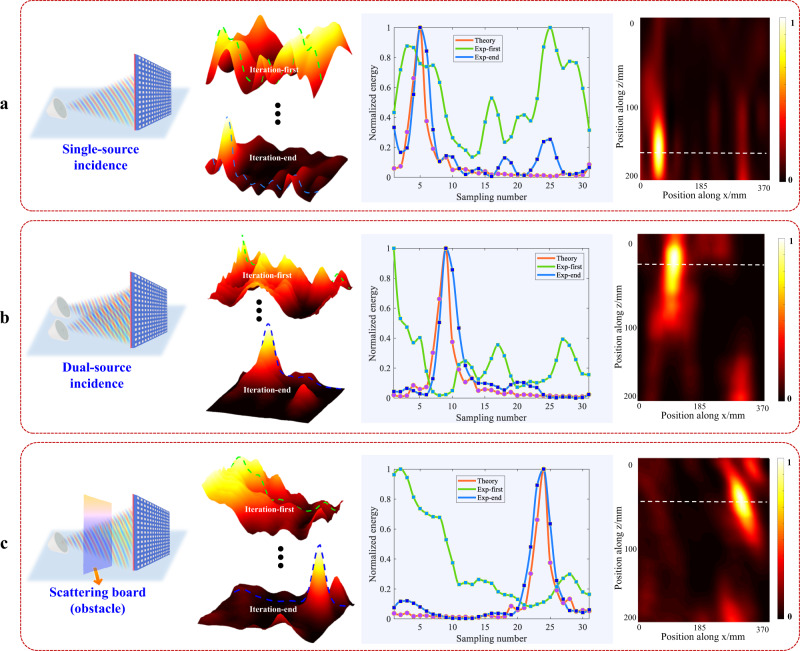
Experimental results for different illuminations and surrounding environments. The red dotted line represents the theoretical focusing effect (one-dimensional curve at the focal position), the green line represents the initial focused electric field during the iterative process of the algorithm, and the blue line represents the focused electric field after the final iteration. **a** Waves from only a single source are incident on the metasurface from an arbitrary angle. The focal position is $(50{mm},175{mm})$. **b** Two sources illuminate the metasurface, and the focal position is $(90{mm},19mm)$. **c** A scattering object is present between the source and the metasurface, and the focal position is $(320{mm},48{mm})$.

In the experiments, we probed only one-dimensional electric field data (31 locations), which are sufficient to achieve accurate adaptive focusing. To verify the focusing effectiveness, we remeasured the two-dimensional electric field data for the initial and final iterations. Note that during the experimental measurements, all incident information were completely unknown to the neuro-metasurface and the optimization algorithm. Figure 4 shows the focusing results for different EM environments (a single source, two sources, and scattering scenario). The focusing efficiencies of the three cases are: 45%, 39%, and 42%, respectively (Supplementary Note 4 describes the method for calculating the focusing efficiency)^34^. The total number of iterations for the three cases were 25, 102, and 72, respectively. Additional 1D and 2D iterative procedures, including various situations, such as different incidence angles, different focal positions, and multiple sources, are provided in Supplementary Notes 4 and 5. The third column shows the normalized energy distributions across different probe positions (horizontal axis of the focus position). The red solid line represents the theoretical electric field data at the focal position, whereas the green and blue solid lines represent the initial field under random voltages and the final field after a series of iterations, respectively. The final effective correlation coefficients in these three scenarios were 90.15%, 86.17%, and 90.2%, respectively. Furthermore, the 2D field distribution of the results shown in the last column shows that the energy is well focused at the specified position.

Notably, there might be multiple focal points (see Supplementary Note 6) since we make use of only one-dimensional electric field data and actively disregard other 2D information on the focal plane. Nevertheless, the SELAF neuro-metasurface can ensure that the energy in the main lobe is always the largest, while the proportion of energy in the side lobes is less than 0.3. Moreover, the abovementioned multi-focus problem can be avoided if 2D electric field data are used as the input to the focus steering network (see Supplementary Note 7).

## Discussion

In conclusion, inspired by the natural focusing ability of the human eye, we proposed the concept of supervised evolving learning and designed a corresponding SEL-driven adaptive focusing system based on a high-transmittance neuro-metasurface. Guided by an evolving focus steering network, the neuro-metasurface exhibits an effective and powerful adaptive focusing ability based on one-dimensional electric field data without human intervention. Experimental results indicate that the proposed SELAF neuro-metasurface is highly adaptable when confronted with complex EM environments. Hence, the SELAF neuro-metasurface demonstrates exciting potential at the intersection of classical EM theory and machine learning. Additionally, the proposed SEL framework not only paves the way for the realization of fast and adaptive focusing but also provides a general adaptive architecture to address more challenging problems. This is only one example of how it is primarily nature’s designs that inspire bionic system design. If we can decode more of these designs, the synergy of human creativity in materials and methodology with nature’s wisdom will offer incredible potential.

## Methods

### The principle of focusing

A focusing-capable neuro-metasurface is composed of many adjustable unit cells, each of which provides a different local transmission spectrum, thereby generating scattered waves. The phase shift of each cell of the metasurface was adjusted by modifying the voltage applied to each cell such that the EM waves transmitted from all the units had the same phase at the focal point, that is, the energy on each path created constructive interference at the focus. Here, we denote the focal point located behind the metasurface by $\left(x,F\right)$, where $x$ is the horizontal coordinate and $F$ is the vertical coordinate and focal length. If the focus holds, the phase of unit cell$i$ should satisfy^15–18,35^:

1 $${\varphi }_{m}^{\left(i\right)}\left(\lambda \right)=\frac{2\pi }{\lambda }\left(\sqrt{{\left(x-{x}_{i}\right)}^{2}+{F}^{2}}-F\right)+{{{{{{\rm{\varphi }}}}}}}_{{shift}}\left(\lambda \right)$$

where $\lambda$ is the wavelength; $F$ is the focal length; and ${{{{{{\rm{\varphi }}}}}}}_{{shift}}\left(\lambda \right)$ is the additional phase shift when applied to a wideband focus, which exhibits an inversely linear relationship with the wavelength $\lambda$, as shown in the following equations:

2 $${{{{{{\rm{\varphi }}}}}}}_{{shift}}\left(\lambda \right)=\frac{a}{\lambda }+b,$$

3 $$a=\delta \frac{{\lambda }_{\min }{\lambda }_{\max }}{{\lambda }_{\max }-{\lambda }_{\min }},$$

and

4 $$b=-\delta \frac{{\lambda }_{\min }}{{\lambda }_{\max }-{\lambda }_{\min }}.$$

where ${\lambda }_{\min }$ and ${\lambda }_{\max }$ denoting the minimum and maximum wavelengths in the band of interest respectively, and $\delta$ being the coefficient for wideband adjustment.

### Focus steering network (FSN)

The accuracy of the FSN, which provides the steering phase shift, is the key to the adaptive focusing system. For the adaptive focusing system, the outgoing focus phase is constant for incoming waves in any incident direction when the focal position is fixed. In accordance with this characteristic, we build the FSN as a mapping from the detected electric fields ${e}_{t}$ to the focus phase compensation ${\varphi }_{t}$ for all unit cells:

5 $$\triangle {\varphi }_{t}=\left[\triangle {\varphi }_{t}^{\left(1\right)},\ldots,\triangle {\varphi }_{t}^{\left(i\right)},\ldots,\triangle {\varphi }_{t}^{\left(N\right)}\right]={f}_{\theta }\left({e}_{t}\right),$$

where $\theta$ denotes the network parameters of the FSN, and ${\triangle \varphi }^{\left(i\right)}$ is the focus phase compensation for unit cell $i$ that satisfies Eqs. (1) and (5).

Considering the visual information processing properties of the human eye, the FSN is implemented with the basic architecture of a convolutional neural network (CNN). CNNs are inspired by the visual cortex and imitate the signal processing of the optical nerve. A CNN consists of an input layer, hidden layers and an output layer, where the hidden layers include layers that perform convolutions. Each convolutional neuron processes data only for its receptive field, and the convolution operation generates a feature map, which in turn is used as input to the next layer. A convolutional layer I s usually followed by other layers, such as a pooling layer and a fully connected layer. In addition, CNNs usually contain nonlinear activation functions, such as the rectified linear unit (ReLU) function, which introduce nonlinearity into the decision function and into the overall network without affecting the receptive fields of the convolutional layers. To simplify the system, the input to the FSN is deemed to consist of one-dimensional electric fields, which are sufficient to accurately predict ${\varphi }_{t}$. The details of the FSN design are given in Supplementary Note 8.

For offline training, we first formed a dataset by generating 2 million data points, where the electric fields were generated with random phases. We then pre-process the original dataset with shuffling and normalization accelerate the convergence of the training process. Subsequently, we divided the pre-processed dataset into a training set (80%) and a test set (20%). In addition, the loss function was defined as the MAE between the output and expected phase compensations (Supplementary Note 8). After training, the loss values on both the training set and the test set were close to 0, indicating that the network showed good prediction performance (see Fig. 3d, e).

### SEL-based focus controller

Once the desired phase compensation has been determined, we modify the voltages applied to the diodes to realize phase compensation for the metasurface units. Here, we acquire the corresponding phase compensation based on the response curve of voltage versus phase, which is easy to obtain based on the design of the unit cell. However, the EM response of the same unit structure will be different for different processing technologies and even under different environmental conditions (e.g., temperature and humidity), which means that we cannot exploit the curve alone to obtain accurate voltages.

By deriving the phase–voltage function, we can obtain the corresponding gradient ${g}_{t}$, i.e., the phase shift produced by the unit cell under a given voltage. Here, ${g}_{t}$ can be acquired either via curve fitting followed by derivation or directly via numerical derivation. With the current phase compensation ${\varphi }_{t}$ and voltage ${u}_{t}$, we can obtain the voltages to be subsequently applied by utilizing the adaptive gradient descent algorithm, Adam^36^:

6 $${u}_{t+1}={u}_{t}-\frac{\alpha {m}_{t}}{1-{\beta }_{1}^{t}}{{\cdot }}\frac{\sqrt{1-{\beta }_{2}^{t}}}{\sqrt{{v}_{t}}+\epsilon \sqrt{1-{\beta }_{2}^{t}}},$$

where $\alpha,{\beta }_{1},{\beta }_{2},$ and $\epsilon$ are algorithm parameters, and the calculations of ${m}_{t}$ and ${v}_{t}$ are represented in Fig. 2d. To overcome the uncertainty of the curve, we can use a fuzzy gradient to compute the voltage, which means that the gradient in Eq. (6) is realized by sampling the normal distribution ${\hat{g}}_{t} \sim N\left({g}_{t},{{{{{{\rm{\sigma }}}}}}}^{2}\right)$. During the iterative calculations, we will store the corresponding ${u}_{t},{e}_{t},{\triangle \varphi }_{t},$ and the last focusing voltage ${u}^{*}$. Notably, the initial voltage is selected using the nearest neighbor rule, namely, the voltage from the historical episode in which the focal position was closest to the current focal position is chosen. Simulations verify the effectiveness and robustness of the proposed SEL architecture (Supplementary Note 9). Throughout the whole process, the effective correlation coefficient, main lobe energy, and side lobe energy are the three criteria for terminating the iteration (Supplementary Note 10).

For a discrete phase–voltage scenario, after the phase compensation has been predicted, the voltage adjustment can be determined only by means of interval discrimination (a piecewise function). For a 2-state diode, we can only flip the voltage of the diode, which results in a 180° phase change. Here, considering the uncertainty of the FSN, we apply a positive voltage to the unit cell with the following probability:

7 $$p\left(x\right)=\frac{{e}^{\delta x}}{1+{e}^{\delta x}},$$

where $x=\triangle {\varphi }_{t}-\pi$, and $\delta > 0$ is a sigmoid coefficient that is determined by the accuracy of FSN. The higher the accuracy of the FSN is, the larger the value $\delta$, and the lower the accuracy of the FSN is, the closer $\delta$ is to 0. That is, the $\delta$ value varies throughout the retraining of the FSN.

## Supplementary information

Supplementary Information

## Data Availability

All other data are available from the corresponding authors on request. Source data are provided with this paper.

## Source data

Source Data

## Code availability

Code used in this work is available from the corresponding authors on request. Some of the code supporting the results of this study are provided as Source Data File, including the loss function for network training and 1-D results of the experiments.
