# Supplementary material for: Eye accommodation-inspired neuro-metasurface focusing
Source: Nat Commun. 2023 Jun 6;14:3301. doi: 10.1038/s41467-023-39070-8 (PMC10244437; doi:10.1038/s41467-023-39070-8)
Supplement: Supplementary file 1 — Supplementary Information [file 41467_2023_39070_MOESM1_ESM.pdf]

Supplementary Information

Eye accommodation-inspired self-adaptive metasurface focusing

Huan Lu[1,3,4,5†], Jiwei Zhao[2†], Bin Zheng[1,3,4,5*], Chao Qian[1,3,4,5*], Tong Cai[1,6*], Erping Li[1,3,4,5], & Hongsheng Chen[1,3,4,5*]

[1]*Interdisciplinary Center for Quantum Information, State Key Laboratory of Extreme Photonics and Instrumentation, ZJU-Hangzhou Global Scientific and Technological Innovation Center, Zhejiang University, Hangzhou 310027, China.*

[2]*School of Electronic Science and Engineering, Nanjing University, Nanjing, 210023, Jiangsu Province, China*

[3]*International Joint Innovation Center, The Electromagnetics Academy at Zhejiang University, Zhejiang University, Haining 314400, China*

[4]*Key Lab. of Advanced Micro/Nano Electronic Devices & Smart Systems of Zhejiang, Jinhua Institute of Zhejiang University, Zhejiang University, Jinhua 321099, China*

[5]*Shaoxing Institute of Zhejiang University, Zhejiang University, Shaoxing 312000, China*

[6]*The Air and Missile Defend College, Air force Engineering University, Xi'an 710051, China*

[†]*These authors contributed equally: Huan Lu, Jiwei Zhao.*

The PDF file includes:

- Supplementary Note 1: Feeding of the active metasurface
- Supplementary Note 2: Design of the unit cell
- Supplementary Note 3: Metasurface function – focusing at different angles and frequencies
- Supplementary Note 4: Experimental results – other incidence environments
- Supplementary Note 5: Experimental results – a single incident horn antenna
- Supplementary Note 6: Issue on multiple focus points
- Supplementary Note 7: Iterative process for 2D electric field data
- Supplementary Note 8: Architecture of the focus steering network (FSN)
- Supplementary Note 9: Simulation verification of the SEL architecture
- Supplementary Note 10: Iteration termination condition

● Supplementary Note 1: Feeding of the active metasurface

Figure S1 shows a diagram of the metasurface power supply board and a schematic diagram of the metasurface power supply. In this experiment, an EVAL-AD5535BSDZ power supply board and an EVAL-SDP-CS1Z control board were used, as shown in Fig. S1a. The EVAL-SDP-CS1Z board allows the EVAL-AD5535BSDZ evaluation board to be controlled via the USB port of a PC using the AD5535B evaluation board software (Fig. S1b). The EVAL-AD5535BSDZ requires 60 V and 5 V external power supplies.

Figure S1c shows a schematic diagram of the metasurface circuit. The structure has a total of 32 power sockets, one of which is the reference ground, and the other slots are provided by the AD5535B board with positive and negative voltages (the voltage of the reference ground is the reference voltage). Since the power supply board can provide only voltages from 0 V to 200 V, the reference ground voltage of the metasurface is set to 5 V here, and the switching states of the diodes can be controlled by simply providing 0 V or 10 V voltages to the other slots.

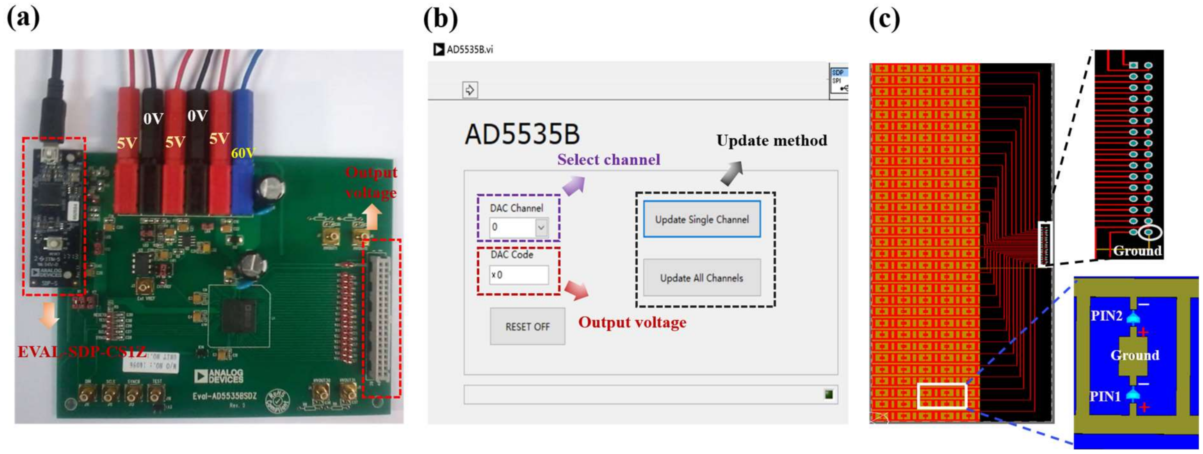

Fig. S1. a, EVAL-AD5535BSDZ board. **b**, The control software. **c**, Diagram of the metasurface power supply circuit.

● Supplementary Note 2: Design of the unit cell

The electromagnetic response of the unit cell of the proposed programmable metasurface was fully analysed via full-wave numerical simulations using the commercial software CST Microwave Studio. For practical realization in the microwave frequency band, M/A-COM MA4FCP305 flip chip PIN diodes, which have a resistance of 1.7 Ω in the on state and a capacitance of 50 fF in the off state, were selected to control the transmission phases. The basic unit cell of the dynamic focusing metasurface is illustrated in Fig. S2.

The unit cell consists of two dielectric layers ($\varepsilon_r = 2.65$) and a bounding layer ($\varepsilon_r = 4.4$). The PIN diodes are welded to the first metal frame of the unit cell, and the positive pole of PIN diode 1 and the negative pole of PIN diode 2 are grounded through a 0.3 mm via hole, as shown in Fig. S2b. The '+ - + -' connection method can ensure that only one diode is in the conducting state each time power is supplied. The dimensions labelled in the figure are $p = 12\ mm$, $d1 = 1\ mm$, $d2 = 0.2\ mm$, $px = 10\ mm$, $py = 7.58\ mm$, $w0 = 1.59\ mm$, $w1 = 1.21\ mm$, $w2 = 2\ mm$, and $w3 = 2.526\ mm$.

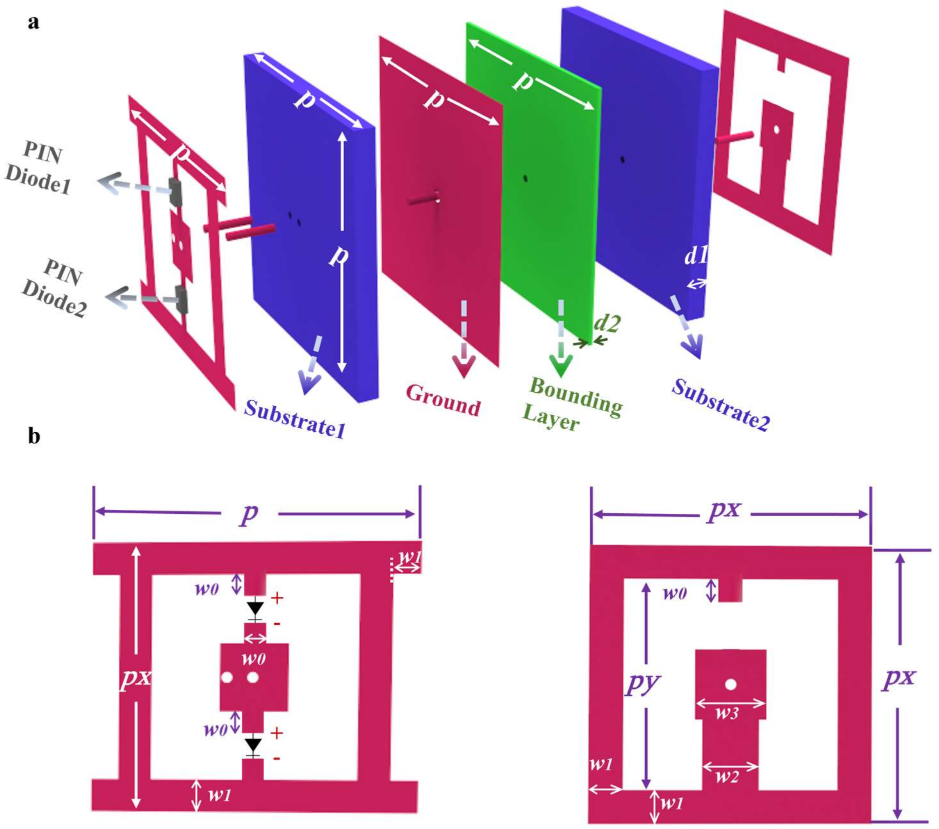

Fig. S2. The unit cell. **a**, Complete structure. **b**, Top and bottom metal frames.

The designed unit cell has high transmittance under different incidence angles and frequencies. Taking a frequency of 5.9 GHz as an example, Table S1 shows the transmission coefficients and transmission phases of the structure under excitation at different incidence angles. In the case of oblique incidence from -50° to 50°, when the two diodes are in different switching states, their transmission coefficients are both approximately 0.95, and the transmission control phase is approximately 180°. Figure S3 shows the transmission coefficients and surface current distributions of the unit cell under different switching states.

Table S1. Amplitude and phase values of the unit structure with different incidence angles at 5.9 GHz

Incidence angle (°)	-50	-40	-30	-20	-10	0	10	20	30	40	50
Transmission coefficient in state 1	0.96	0.95	0.96	0.96	0.97	0.97	0.98	0.96	0.95	0.95	0.96
Transmission coefficient in state 2	0.96	0.95	0.96	0.96	0.95	0.96	0.97	0.96	0.95	0.94	0.94
Phase (°) in state 1	-249	-260	-274	-283	-295	-299	-300	-298	-296	-292	-290
Phase (°) in state 2	-90	-98	-105	-112	-115	-119	-116	-112	-105	-95	-86

a

b

c

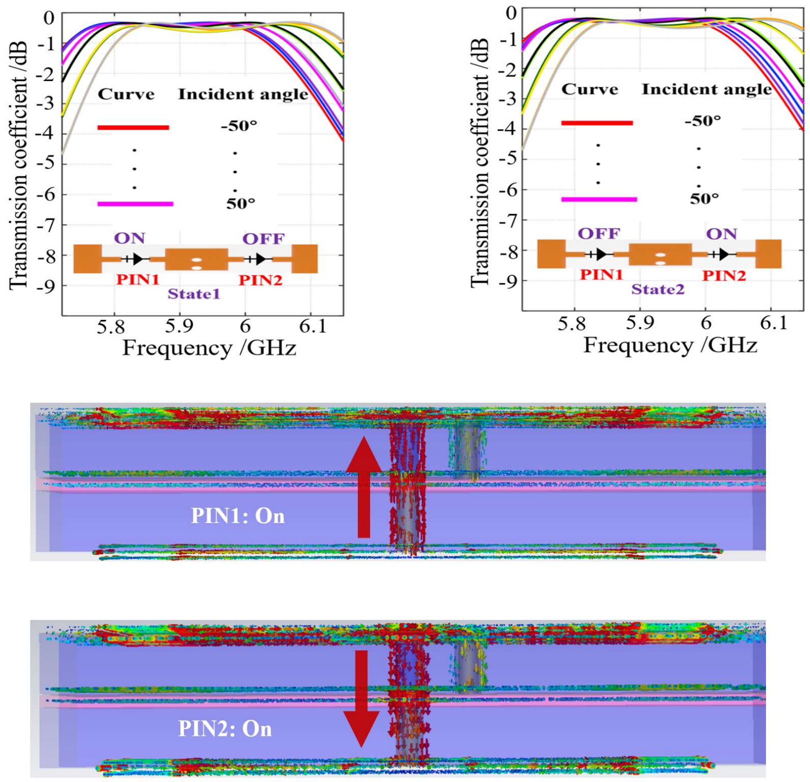

Fig. S3. a, Transmission coefficients at different frequencies and incidence angles. **b, c** Surface current distributions under different states of the PIN diodes.

- ## Supplementary Note 3: Metasurface function – focusing at different angles and positions

We used the selected frequency (5.9 GHz) as an example to demonstrate the focusing performance using binarized phases. The compensated phases at different locations on the metasurface can be calculated using the following formula (explained in the manuscript):

$$\varphi_m^{(i)}(\lambda) = \frac{2\pi}{\lambda}\left(\sqrt{(x - x_i)^2 + F^2} - F\right) + \varphi_{shift}(\lambda).$$

Calculation of $\varphi_m^{(i)}(\lambda)$ based on the phase and direction of the incident wave follows optical principles. This approach requires a prerequisite that the phase and direction of the incident wave are known, which may limit its applicability in situations involving multiple wave sources, obstacles, and other similar environments. However, to clarify the focusing ability of the two phases, we assumed that the number of unit cells was 31, and the focusing position was at a distance of 100 mm directly above the metasurface ($F = 100$). The EM wave was incident perpendicular to the metasurface, and there was no obstacle. First, we calculated the theoretical compensation phase using the above equation, as indicated by the blue curve in Fig. S4a. Because our structure can achieve only two phases (0° and 180° or a certain phase shift), we binarized the compensation phase to obtain the phase when the metasurface operated (red curve in Fig. S4a). It can be observed that there is a deviation between them in Fig. S4 a. Rayleigh–Sommerfeld diffraction theory (Supplementary Note 8) was used to verify the focusing effect. Figure S4 b shows the focusing effect that satisfies the theoretical and binarized phases. Although the focusing efficiency was reduced, the binarized phase still demonstrated the feasibility of focusing.

The focusing effect was simulated using CST2020 at different incidence angles and positions, as shown in Fig. S4d. Under the normal incidence condition ($\theta = 0°$), the three random focus positions are: (250 mm, 80 mm), (60 mm, 80 mm), and (330 mm, 80 mm). Under oblique incidence conditions ($\theta = 30°$), the three random focus positions are (250 mm, 100 mm), (60 mm, 100 mm), and (330 mm, 100 mm). This can be observed in Fig. S4d that the tunable transmission structure exhibits good focusing characteristics at different positions and incidence angles.

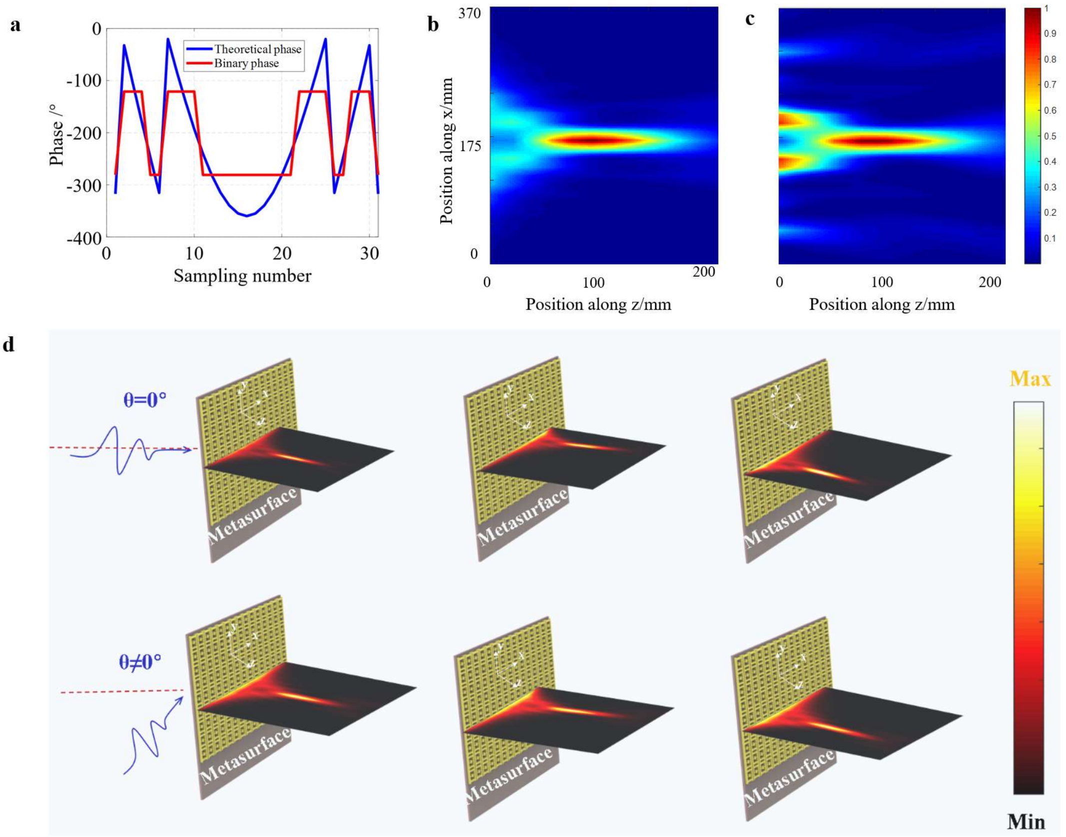

Fig. S4. a, Comparison of theoretical phase and binary phase. **b,** The focusing effect using theoretical phase. **c.** The focusing effect using binarized phase. **d,** Metasurface focusing effect in other positions.

- ## Supplementary Note 4: Experimental results – other incidence environments

Figure S5 shows the magnitude, real part, and phase information of the electric field on a spatial plane ($200\ mm\ \times\ 400\ mm$) as measured under different incidence environments. When there is only one horn antenna as the source of the incident wave (Fig. S5a), the overall amplitude of the plane wave is relatively uniform, and the real part and phase are smooth. When there are two horn antennas as the sources (Fig. S5b), due to the coupling of the dual sources and the superposition in space of the electromagnetic waves emitted by the two antennas, the measured electric field information is relatively chaotic. In Fig. S5c, a scattering plate is added as interference between the single horn antenna and the metasurface. The incident electromagnetic wave passes through the scattering medium before reaching the metasurface; consequently, the amplitude, phase, and real part of the spatial electric field change, and it no longer has a uniform plane wave shape. Figure S5d shows the four-port vector network analyser, which can be used as a signal source output while collecting probe information. Figure S5e shows the plate used as the scattering medium, which consists of two layers, with total side lengths of $500\ mm \times 500\ mm$ and a thickness of 3 mm. The outer layer, layer 1, is a laser-scattering

acrylic material with a relative dielectric constant of 2.6, and the surface is designed to be uneven to effectively scatter and modulate electromagnetic waves. Layer 2, the inner layer, is a dielectric plate designed in the shape of a cat, and its relative permittivity is 4.4. In addition, some irregular metal copper foils are pasted on layer 2 to obstruct electromagnetic waves.

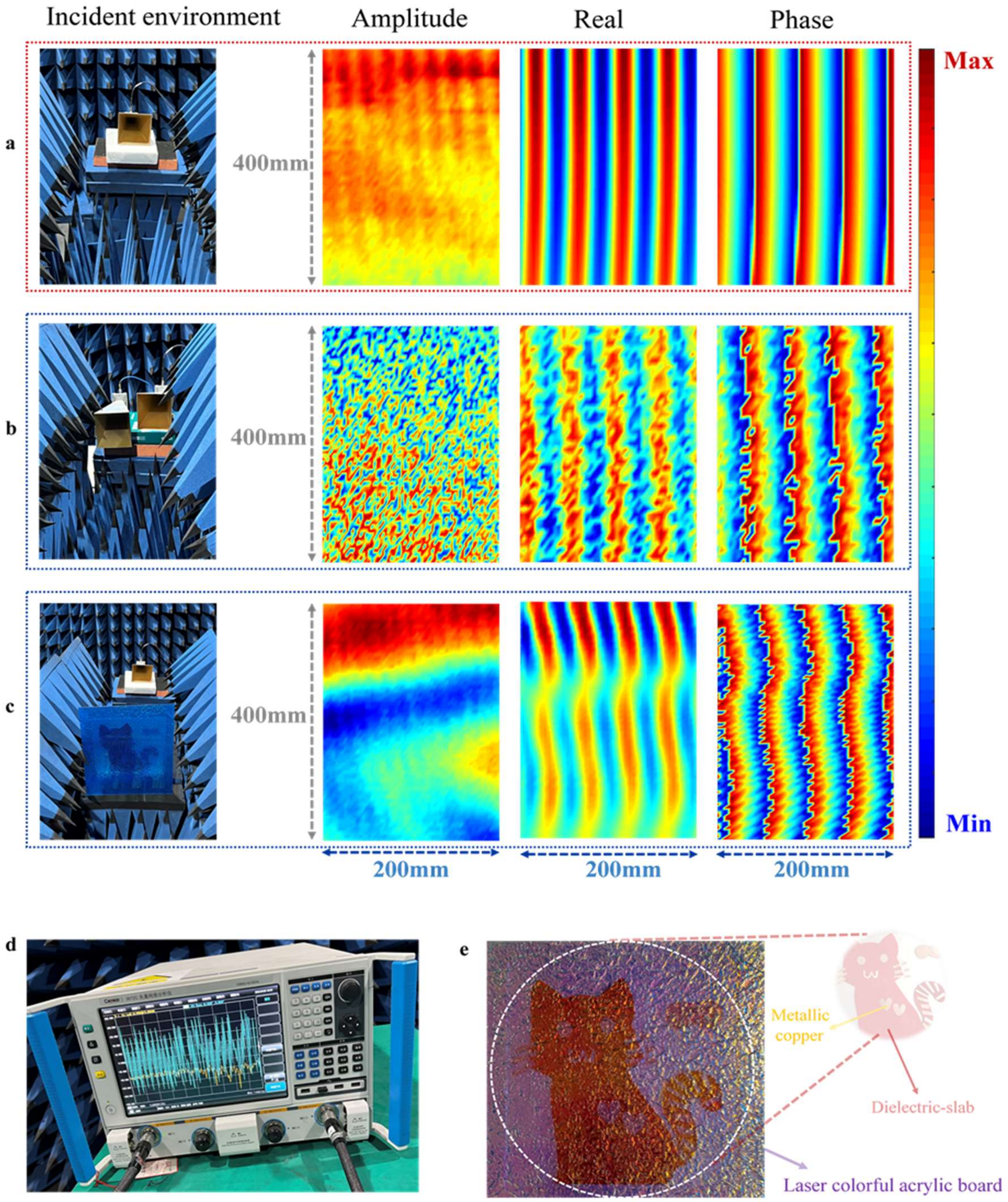

Fig. S5. Measured electromagnetic wave amplitudes, real parts, and phases under different incidence environments. **a**, Only one incident source. **b**, Two incident horn antennas. **c**, A scattering medium lying between the single horn antenna and the metasurface. **d**, Vector network analyser with four ports. **e**, Scattering plate.

Figure S6 shows the iterative processes for different positions in a multisource electromagnetic environment. N_{focus_i} represents the total number of iterations for each data point. We quantitatively describe the focusing effect in terms of focusing efficiency.

In principle, the efficiency of the transmittance neuro-metasurface is calculated using the ratio of the power at the focus position and input power. This ratio is represented by $\eta_{foc} = \frac{P_{foc}}{P_{inc}}$. The formula for calculating $\frac{P_{foc}}{P_{inc}}$ is defined as $\frac{P_{foc}}{P_{inc}} = \frac{\oint |\vec{E}|^2 ds_1}{\oint |\vec{E}|^2 ds_2}$, where s_1 and s_2 represent the areas occupied by the focal spot and incident beam, respectively. Furthermore, we measured the $|\vec{E}|^2$ distribution in the focal plane (the xz-plane). Simultaneously, two integrations ($\oint |\vec{E}|^2 ds_1$, $\oint |\vec{E}|^2 ds_2$) were evaluated using the measured electric-field patterns, where the first and second integrations represent the areas occupied by the focal spot and entire region occupied by the metasurface, respectively. The radius of the focal spot power was determined using the formula, $R = 4\lambda M^2 F/(\pi D)$, where $M = 1$, D is the size of the metasurface, and F is the focal length. Based on these calculations, the evaluated focusing efficiencies of the three cases are: 33%, 39%, and 32%, respectively.

The curves (Figs. S6a, b, and c) show the one-dimensional focusing process. To visualise the dynamic focusing process, we remeasured the corresponding two-dimensional electric field (Figs. S6d, e, and f). In Fig. S6a, the focal position was$(180\,mm, 170\,mm)$, and the total number of iterations, N_{focus_1} was 120. In Fig. S6b, the focal position was$(90\,mm, 19\,mm)$, and the total number of iterations, N_{focus_2} was 102. In Fig. S6c, the focal position was$(310\,mm, 22\,mm)$, and the total number of iterations N_{focus_3} was 105.

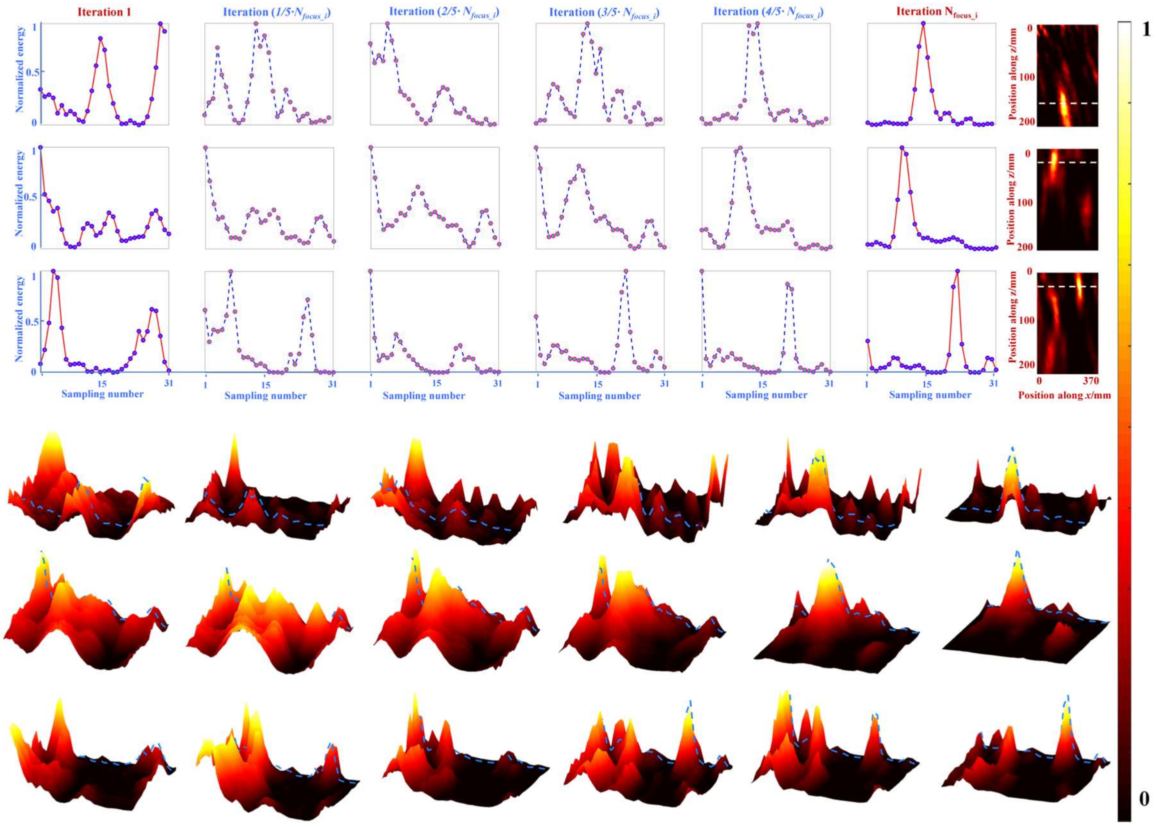

Fig. S6. Dynamic focusing processes at different positions under dual-source incidence conditions. Panels **a**, **b** and **c** show the one-dimensional focusing processes. Panels **d**, **e** and **f** show the two-dimensional focusing processes corresponding to **a**, **b** and **c**.

Similarly, Fig. S7 shows the dynamic focusing results when an obstacle is placed between the emission source and the metasurface. The focusing efficiencies in these three cases were 42%, 37%, and 35%, respectively. The curves (Fig. S7a, b, and c) show the one-dimensional focusing process. To visualise the dynamic focusing process, we remeasured the corresponding two-dimensional electric field (Fig. S7d, e, and f). In Fig. S7a, the focal position was$(320\ mm, 48mm)$, and the total number of iterations N_{focus_4} was 72. In Fig. S7b, the focal position was$(80\ mm, 80\ mm)$, and the total number of iterations N_{focus_5} was 47. In Fig. S7c, the focal position is $(65\ mm, 20\ mm)$, and the total number of iterations N_{focus_6} was 38.

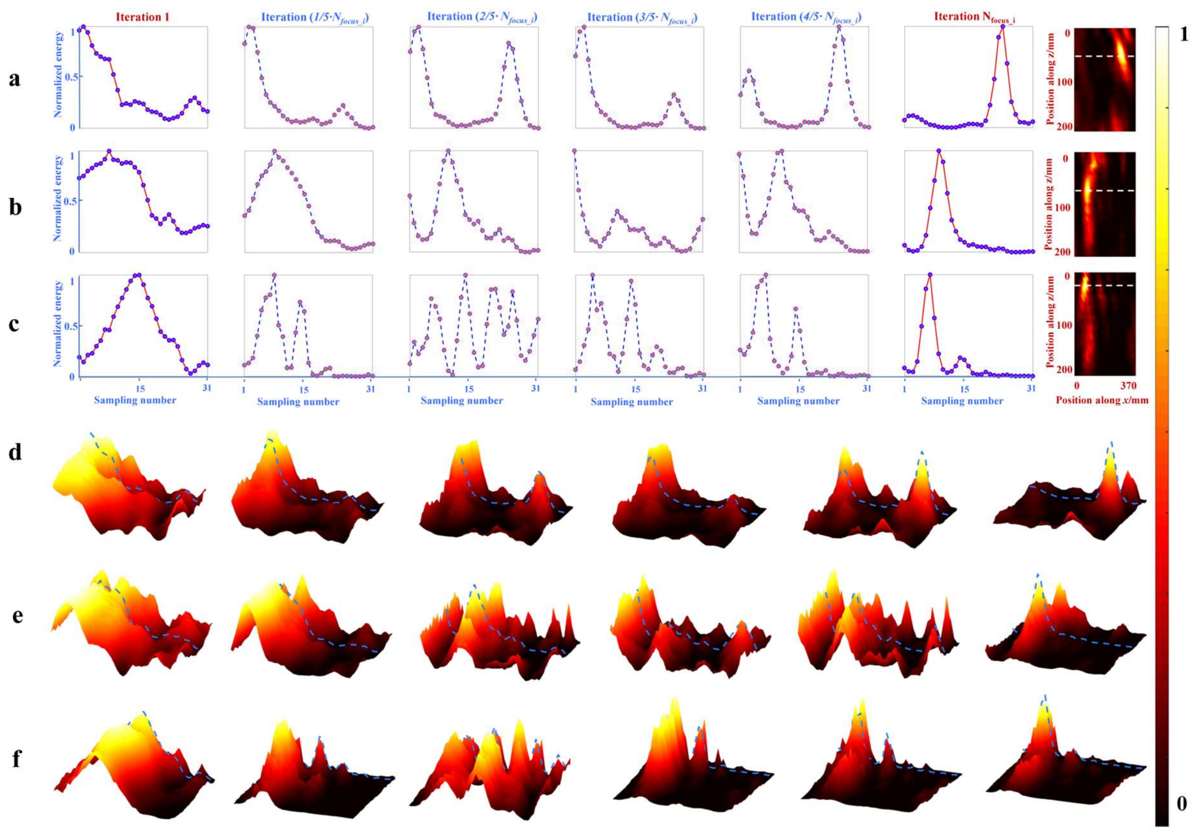

Fig. S7. Dynamic focusing processes at different positions in a complex electromagnetic environment created by a single horn antenna and a scattering medium. Panels **a**, **b** and **c** show the one-dimensional focusing processes. Panels **d**, **e** and **f** show the two-dimensional focusing processes corresponding to **a**, **b** and **c**.

● Supplementary Note 5: Experimental results – a single incident horn antenna

Figure S8 shows the iterative processes when focusing at different positions with different angles of incidence. The focusing efficiencies of the five cases are: 45%, 39%, 37%, 33%, 46%. N_θ uniformly represents the total number of iterations for each data point. In Fig. S8a, the incidence angle is 0°, the focal position is $(50mm, 175mm)$, and the total number of iterations $N_{\theta=0^\circ}$ is 25. In Fig. S8b, the incidence angle is 10°, the focal position is $(90mm, 93mm)$, and $N_{\theta=10^\circ}$ is 16. In Fig. S8c, the incidence angle is 20°, the focal position is $(185mm, 105mm)$, and $N_{\theta=20^\circ}$ is 31. In Fig. S8d, the incidence angle is 30°, the focal position is $(270mm, 50mm)$, and $N_{\theta=30^\circ}$ is 40. In Fig. S8e, the incidence angle is 40°, the focal position is $(120mm, 85mm)$, and $N_{\theta=40^\circ}$ is 23. In addition, to illustrate the iterative process more clearly, we measured the two-dimensional electric field corresponding to the one-dimensional data at the same time, and the resulting electric field energy diagrams are shown in Fig. S9. Note that the two-dimensional

electric field information is provided here only for visualization of the training results and was not used as training data.

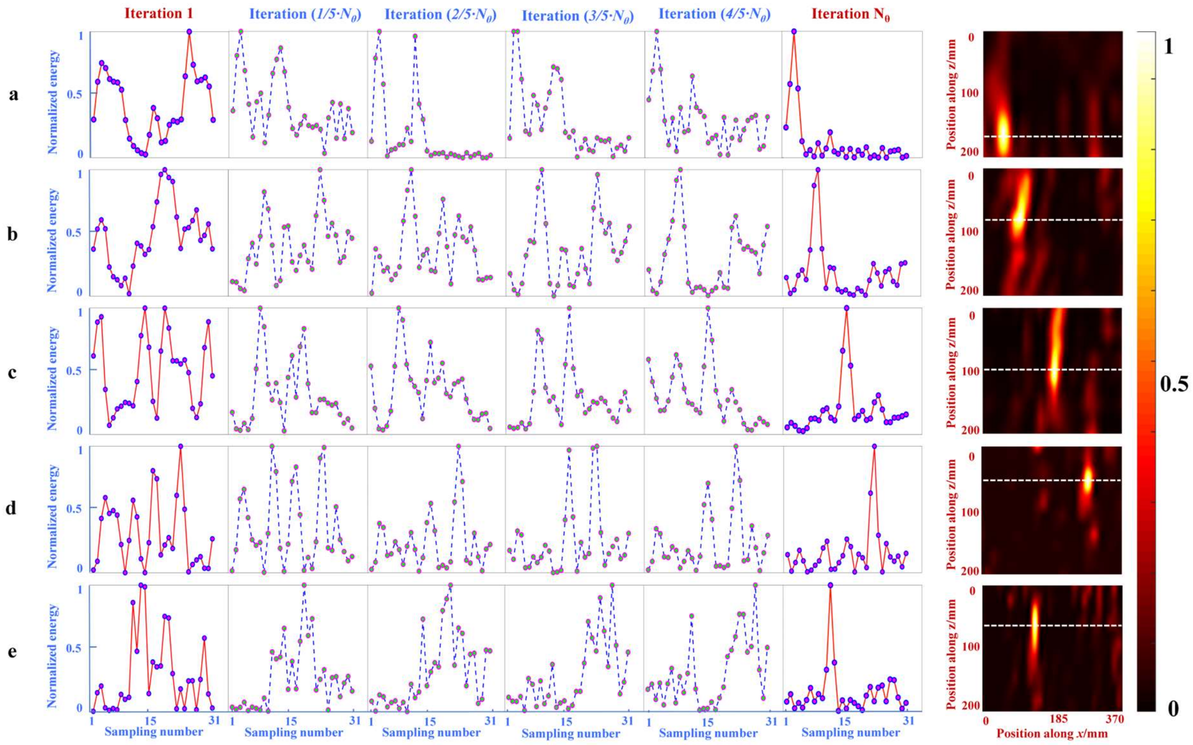

Fig. S8. Iterative processes of dynamic focusing with different focal points.

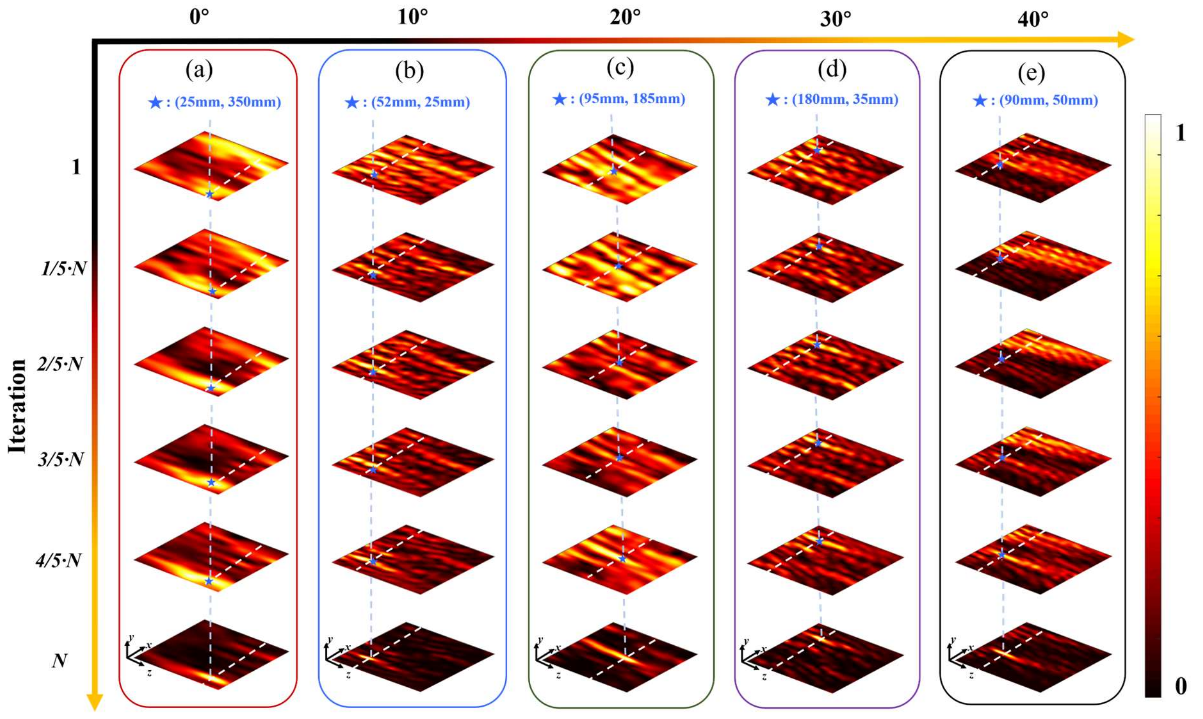

Fig. S9. Two-dimensional electric fields corresponding to **Fig. S8**.

● Supplementary Note 6: Issue of multiple focal points

Since the algorithm only considers one-dimensional data information, it cannot consider the two-dimensional electric field of the entire observation plane; consequently, multi-focus problems may occur, as shown in Fig. S10.

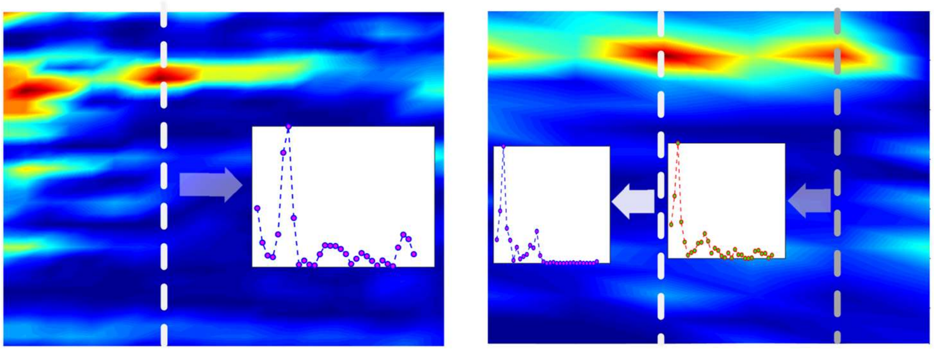

Fig. S10. Multi-focus situation.

● Supplementary Note 7: Iterative process for 2D electric field data

If as much information as possible can be obtained, the problem shown in Fig. S10 can be solved. For verification, we trained a network model taking two-dimensional electric field data as input. The network model with two-dimensional input had the sufficient spatial fitting ability to finally achieve focusing at the target position after iterating 23 times. If only one-dimensional electric field information is used, the model will stop after 20 iterations. However, when observing the entire two-dimensional field diagram, we find that although focusing at the target position is achieved at this time, there is also an energy concentration at the position indicated by the white dashed ellipse (Fig. S11).

Although two-dimensional electric field data can be used to better predict the target compared with one-dimensional data, the two-dimensional data acquisition cycle is long and the network training speed is slow; hence, this approach is not the focus here for the time being. Indeed, we can achieve the goal relatively quickly and accurately with only 31 data points.

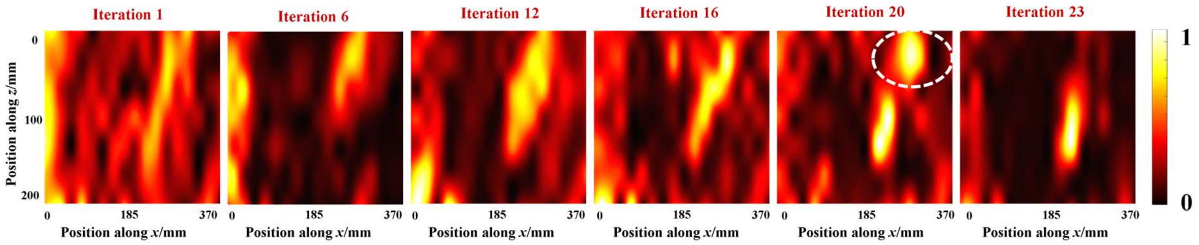

Fig. S11. Adaptive focusing of a two-dimensional electric field under normal incidence.

- **Supplementary Note 8: Architecture of the focus steering network (FSN)**

1) Data generation and training environment

In the far field, Rayleigh–Sommerfeld diffraction can be applied for the calculation of the electric field distribution of the metasurface, and this approach was used to generate 2 million sets of training data:

$$U(x_0) = \iint \sum U(x_1) \frac{1}{i\lambda} cos < n, x_{01} > \frac{exp(\, ikx_{01})}{x_{01}} ds \qquad (S1)$$

where $U(x_0)$ and $U(x_1)$ represent the electric fields at point X_0 on the metasurface and point X_1 on the image plane, respectively; $\sum$ is the virtual object region; λ is the wavelength in vacuum; n is a vector oriented perpendicular to the image plane; x_{01} is the distance between X_0 and X_1; and $cos < n, x_{01} >$ is the inclination factor. We assume an object with uniform amplitude and phase, so we can let $U(x_1) = A$. The range of the observation area d is $(0\ mm, 200\ mm]$. Thus, the electric field distribution on the metasurface can be expressed in a simpler form as follows:

$$U(x_0) = \frac{Ad}{i\lambda} \iint \sum \frac{exp(\, ikx_{01})}{x_{01}^2} ds \qquad (S2)$$

2) CNN training

A CNN is composed of an input layer, hidden layers, and an output layer. The hidden layers include layers that perform convolution. Here, we used four convolutional layers and two fully connected layers and used the ReLU activation function for network training. We first formed a dataset by generating 2 million data points, where the electric fields were generated with random phases. We then pre-processed the original dataset with shuffling and normalization to accelerate the convergence of the training process. We subsequently divided the pre-processed dataset into a training set (80%) and a validation set (20%). As the evaluation index for the network's predictive capability, we used the MAE, which is defined as follows:

$$MAE = \frac{1}{n} \sum_{i=1}^{n} |\hat{y}_i - y_i| \qquad (S3)$$

where n represents the number of samples, $\hat{y}_i$ represents the predicted value, and y_i represents

the true value. When the predicted values are completely consistent with the true values, the MAE is equal to 0, indicating a perfect model; the greater the error is, the greater the MAE value.

We used a server running the Ubuntu Linux OS with four 8X Tesla V100 GPUs and 256 GB of total GPU memory. The system supported 96 threads running simultaneously, which greatly saved time for network computations and training. When the number of training epochs was set to 100 and the total data volume was 6 GB, the time to train the network was only approximately 10 minutes.

● Supplementary Note 9: Simulation verification of the SEL architecture

First, we specified the target electric field, recorded as E_{Theory}:$[E_1 \dots E_{31}]$. The SELAF system detects one-dimensional electric field data in a random incidence environment, recorded as E_{Test}: $[E_1 \dots E_{31}]$. E_{Test} and E_{Theor} were compared according to the rules of iteration termination conditions (Supplementary Note 10). After that, the FSN generated the output $\Delta\varphi_t$, and the evolving learning network produced the adjusted voltage matrix ΔU_t. If these outputs fail to meet the required condition, the system underwent a continuous closed-loop adaptive tuning process to enable focusing in a random incidence environment.

The accuracy and robustness of the proposed SEL architecture were verified through simulations. First, Rayleigh–Sommerfeld diffraction theory was used to calculate the electric field when all phases were zero, and this was used as the initial value for the first iteration. Figure S12 shows the iterative processes for different focal positions (indicated by purple asterisks in the figure) when the electromagnetic waves are normally incident. $N_{t(i)}$ uniformly represents the total number of iterations for each data point.

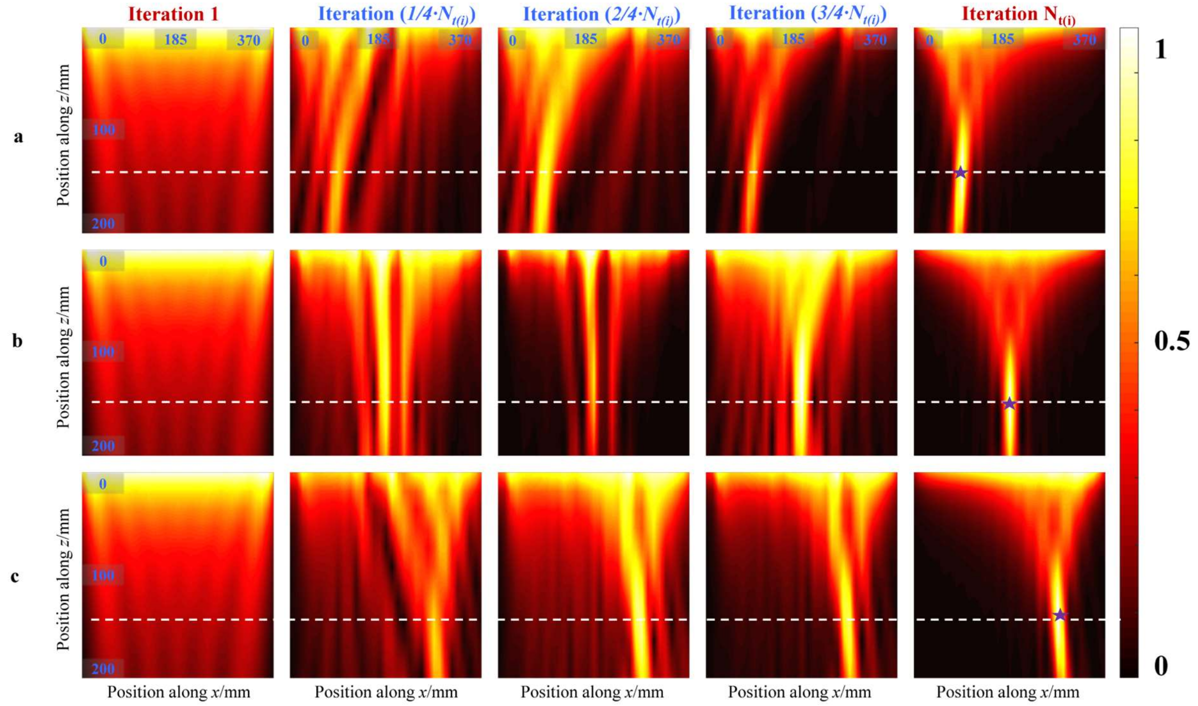

Fig. S12. a, The focal position is (90 mm, 95 mm). **b**, The focal position is (185 mm, 95 mm). **c**, The focal position is (260 mm, 95 mm). $N_{t(a)} = 17$, $N_{t(b)} = 12$, and $N_{t(c)} = 25$.

The SELAF system enables the focus on multi-frequencies. On the one hand, the unit cell we designed can achieve the 180° phase flip over a wide frequency range when the external voltage changes, as shown in Fig. S13 (take the normal incident and other incidence angles as examples). The horizontal coordinate represents the frequency, the vertical coordinate represents the phase, and the purple dashed line represents the range of the frequency bands with magnitudes above -3 dB.

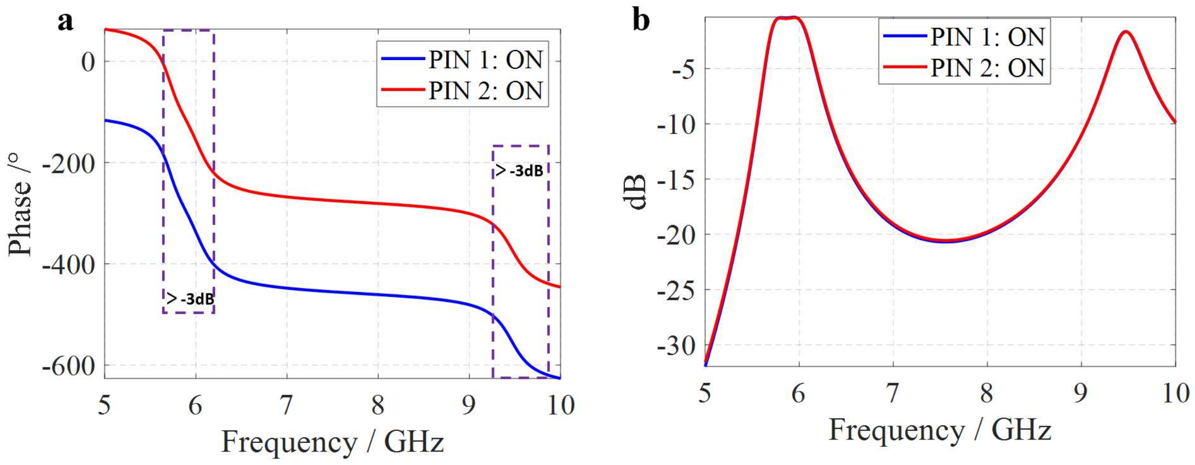

Fig. S13. S-paremeters. **a,** Transmission phase at different frequencies. **b,** Transmission coefficients at different frequencies.

On the other hand, Our method is band-independent and continuously and automatically learns the phase response of the unit cell to achieve different focusing tasks. We must specify the focus positions of different frequencies and use them as optimisation targets for the system simultaneously. Figure S14 shows two groups of focusing results at special positions when the system optimises 6 and 9.5 GHz at the same time. Figs. S14a and b are simultaneously used as the optimisation objectives of the SEL model, and Figs. S14c and d are used as the optimisation objectives of other tasks.

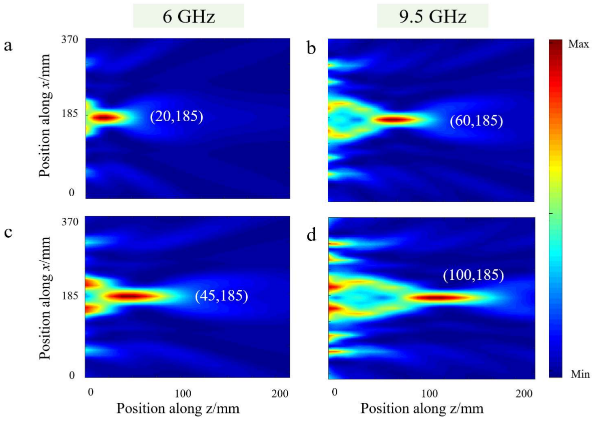

Fig. S14. Multi-wavelengths focusing results. **a** and **b** are a set of tasks optimized simultaneously; c and d are a set of tasks optimized simultaneously.

Although global optimisation can achieve multi-wavelength focusing in some special positions, the focusing effect is less than that of a single frequency point owing to the phase limitation of the unit cell. Figure S15 shows the result of training alone at 9.5 GHz at the focus positions of (100 mm and 185 mm). Comparing Fig. S14d and Fig. S15e, multi-wavelength focusing is achieved at the expense of focusing efficiency. Thus, multi-wavelength focusing is not the key point of our manuscript.

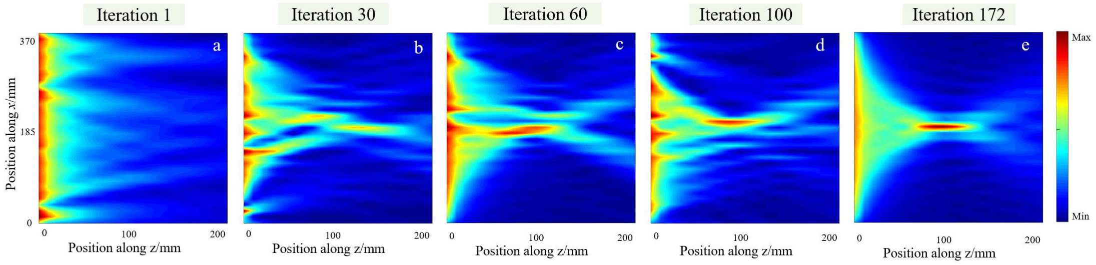

Fig. S15. The iteration process at 9.5 GHz.

● Supplementary Note 10: Iteration termination conditions

Three parameters were set as the collective conditions for termination of the iterative process.

1) Effective correlation coefficient > 85%

Here, the effective correlation coefficient $r(E_{Theory}, E_{Test})$ is defined as the correlation between the tested spot power E_{Test} and the theoretical spot power E_{Theory}, where all values of spot power are normalized. Then, the correlation coefficient can be calculated with the following formula:

$$r(E_{Theor}, E_{Test}) = \frac{C(E_{The}, E_{Test})}{\sqrt{Var[E_{Theor}]Var[E_{Test}]}} > 85\% \qquad (S4)$$

$$\begin{aligned} C(E_{Theory}, E_{Test}) &= E[(E_{Theory} - E[E_{Theory}])(E_{Test} - E[E_{Test}])] \\ &= E[E_{Theor} E_{Test}] - 2E[E_{Test}]E[E_{Theory}] + E[E_{Theor}]E[E_{Test}] \\ &= E[E_{Theory}E_{Test}] - E[E_{Theory}]E[E_{Test}] \end{aligned} \qquad (S5)$$

where $C(E_{Theory}, E_{Test})$ is the covariance of E_{Theory} and E_{Test}, $Var[E_{Theory}]$ is the variance of E_{Theory}, and $Var[E_{Test}]$is the variance of E_{Test}. **E** is the mathematical expectation.

The radius R used to evaluate the spot power is determined by the following equation:

$$R = 4\lambda M^2 f/(\pi D) \qquad (S6)$$

where $M = 1$, D is the diameter of the metasurface, f is the focal length, and λ is the wavelength.

2) Main lobe energy proportion $\rho_m \geq 0.3$

The ratio of the energy in the main lobe (at the focal point), E_{Major_lobe}, to the total measured electric field energy, E_{Test_total}, is required to be greater than 30%:

$$\rho_m = \frac{E_{Major\_lobe}}{E_{Test\_total}} \geq 30\% \qquad (S7)$$

3) Total side lobe energy proportion $\rho_s < 0.3$

The energy ratio of the side lobe, E_{Side_lobe}, to the total measured electric field energy, E_{Test_total}, is required to be lower than 30%

$$\rho_s = \frac{E_{Side\_lobe}}{E_{Test\_total}} < 0.3 \qquad (S8)$$
